# Can Marketization of Environmental Governance Improve Public Health?—Empirical Analysis Based on the Emission Trading System

**DOI:** 10.3390/ijerph192315518

**Published:** 2022-11-23

**Authors:** Yongyan Huang, Shilong Li

**Affiliations:** 1 School of Management Science and Real Estate, Chongqing University, Chongqing 400044, China; 2 Research Center for Construction Economics and Management, Chongqing University, Chongqing 400044, China

**Keywords:** emission trading system, health effects, difference-in-difference model, environmental health equity

## Abstract

To deal with the pollution issue caused by long-term economic development, China has introduced a number of environmental governance measures and made some progress. In the context of the strategy of developing a “Healthy China”, the Chinese government proposed to include health in the formulation and implementation of environmental regulation policies, which places a higher demand on the implementation effect of environmental policies. This study attempts to clarify the causal link between market-based environmental governance and public health, and quantify the implementation effects of market-oriented environmental governance, so as to provide accurate assessments for addressing environmental degradation and improving public health. Based on the CHNS database and provincial panel data from 2000 to 2015, this paper constructs a binary logit-based difference-in-difference model to investigate the effects of the emission trading system (ETS) pilot policy on public health measured by the incidence of respiratory diseases, heart disease, and other diseases. The results show that ETS significantly reduces the incidence of respiratory and other diseases but has no significant impact on the incidence of heart diseases. The improvement effects of ETS on public health mainly come from the reduction of SO_2_ under the principle of total volume control. However, heterogeneity analysis reveals that the health-improving effects of ETS are not as expected. Although ETS can enhance the health of vulnerable populations, such as workers near pollution sources and rural residents, it has no discernible impact on the health of those far from pollution sources and urban residents.

## 1. Introduction

Environmental pollution and climate change are two major challenges currently threatening human health [1]. The extensive development pattern of high energy consumption and high pollution in the past has contributed to China’s rapid economic development, but it has also resulted in an unbearable heavy burden on the ecosystem, posing a huge threat to public health [2]. According to the Global Air Quality Report, 9 million people died in 2019 as a result of environmental pollution, of which nearly 75% died from air pollution [3]. As the leading cause of mortality among all environmental problems, air pollution has grown to the fourth largest threat of premature death globally, trailing only hypertension, smoking, and malnutrition [4]. Moreover, air pollution also leads to high social costs. From the perspective of disease cost, the annual economic loss caused by air pollution is estimated to be about 1.2% of China’s annual GDP, while from the perspective of willingness to pay, this economic loss reached as high as 3.8% of annual GDP [5]. The rising incidence of respiratory and cardiovascular diseases caused by air pollution leads to significant health losses and excess disease burden, which seriously lowers people’s quality of life [6,7].

The increasingly serious air pollution problems arising from energy consumption dreadfully endanger public health, and stifle economic and social progress, thus gaining widespread attention from governments and academia around the world. A quantitative study shows that every 1 million tons increase in SO_2_ emission would result in an additional 0.5 and 0.3 lung cancer and respiratory disease deaths, respectively, among 10,000 people [8]. How to effectively reduce the emission of air pollution and protect public health has become the focus of environmental governance policies around the world.

Developed countries such as Europe and the United States have witnessed significant improvements in environmental quality by introducing mandatory controls and market-oriented tools for environmental governance [9]. In contrast, the promulgation and implementation of environmental governance in China are relatively lagging behind, with mandatory environmental regulations dominating and market-based environmental governance tools insufficient [10]. However, the incentive for promoting technological innovation and developing a green economy of mandatory controls is limited, whereas market-oriented instruments can stimulate technological innovation and improve the efficiency of environmental governance. It is critical to deepen the reform of the environmental governance mechanism and emphasize the role of market-oriented instruments in environmental governance. As a market-oriented environmental pollutant control strategy, the ETS has been practiced in China for nearly two decades. The effects of ETS on promoting enterprises’ green technological innovation and reducing the concentration of industrial emissions represented by SO_2_ have been confirmed [11,12]. However, whether this environmental dividend can be converted into public health benefits remains to be further investigated.

In the context of implementing the “Health China” strategy, the capacity to improve public health has become a more stringent requirement for measuring the effectiveness of environmental governance policies. However, the existing literature on ETS focuses primarily on the environmental green dividend and rarely on the public health dividend. In addition, the relevant heterogeneity studies are relatively homogeneous, mainly focusing on improvement effect differences caused by individual physiological, while little attention has been paid to the effects due to differential pollution exposure. Since the purpose of environmental policies is to protect human health, it is necessary to fill such a research gap. For this purpose, this paper takes the pilot of SO_2_ as an example and builds a difference-in-difference model to empirically analyze the effects of marketization of environmental governance on public health and its theoretical mechanism, utilizing the China Health and Nutrition Survey (CHNS) data and provincial panel data. Besides, this paper further explores the differential health effects of environmental governance on different pollution-exposed groups to provide a scientific basis for the formulation of policies related to environmental pollution prevention and environmental health equity in China.

## 2. Literature Review and Theoretical Mechanism Analysis

### 2.1. Literature Review

#### 2.1.1. Environmental Governance and Environmental Pollution

Most studies on environmental governance and environmental pollution conclude that environmental governance forces inefficient enterprises to withdraw from the market or upgrade production technology and pollution control technology in order to achieve emission reduction goals. Zhen et al. [13] investigated the impact of mandatory environmental regulation on enterprises’ environmental performance and found that cleaner production standards policy significantly reduced the SO_2_ emissions’ intensity of the enterprises but failed to reduce the aggregate SO_2_ emissions. Shapiro [14] and Tang [15] explored the impact of pollution tax policies on pollution emissions in the United States and China, respectively, and pointed out that the levy of pollution taxes effectively reduces the emissions of the corresponding pollutants, implying that the marketization of environmental governance can improve environmental quality to some extent. Reviewing the existing literature, it is not difficult to find that both mandatory policies and market-oriented environmental governance policies can alleviate environmental pollution pressure. However, in the long run, to effectively solve the problem of environmental pollution, it is necessary to rely on technological innovation, especially green technology-oriented technological innovation [16]. Although mandatory environmental regulations can achieve significant environmental improvements in the short term, their mandatory regulations also deprive enterprises of incentives to innovate; whereas the innovation compensation effect of market-oriented environmental governance, such as environmental taxation and the ETS, encourages enterprises to adopt green technologies and switch to cleaner production, which can broadly advance social progress and industrial structure modernization, and ultimately improve the environmental quality [17,18,19]. Du et al. [20] found that ETS leads to the reduction of pollution emissions by encouraging enterprises to strengthen cleaner production. Further research by Zhi et al. [21] also indicated that ETS promotes clean energy consumption by improving the production of enterprises.

#### 2.1.2. Environmental Pollution and Public Health

Early studies on the relationship between environmental pollution and public health are mainly conducted in the medical field. These studies have reached a consensus on the deleterious effects of environmental pollution on public health from the standpoint of pathological mechanisms [22,23]. Following the clarification of the adverse effects of environmental pollution, policymakers and researchers are more concerned with how to formulate environmental governance policies to mitigate the harm of environmental pollution on public health. Thus, based on Grossman’s health production function, Cropper [24] and Gerking et al. [25] initiated the study of health economics on environmental pollution and public health by linking air pollution to health. Studies in this field found that increasing air pollution not only causes lower birthweight and poor health in newborns [26] but also increases the likelihood of coughing and asthma in residents [27], thereby increasing mortality [28] and shortening average life expectancies to a certain extent [29]. Additionally, the negative impacts of air pollution on public health have a significant spatial spillover effect because of its high mobility. Chen et al. [30] developed a spatial Durbin model using 116 urban observation samples and discovered that air pollution in nearby cities has a negative impact on public health in local cities.

#### 2.1.3. Environmental Governance and Public Health

Various studies have focused on the relationship between environmental governance and infant mortality due to the vulnerability of infants and the availability of the data. It has been confirmed that environmental governance initiatives such as the Clean Air Act in the United States [31], desulfurization policies in the power sector in Germany [32], the two control zones [33], and SO_2_ ETS [34] in China have significantly reduced infant mortality. With the continuous supplement and expansion of the health database, it has also been found that environmental governance policies can reduce the incidence of diseases associated with environmental pollution. The implementation of China’s “Ten Atmospheric Regulations” and carbon emission trading system, for example, can reduce the emissions of PM_2.5_ and the mortality of diseases caused by PM_2.5_ [35,36]. Most recently, research on ETS and public health from the perspective of environmental governance marketization are gradually emerging. Guo et al. [37] took the carbon emissions trading policy as an example and found it can promote the improvement of residents’ health. While some studies used “carbon emission trading volume” as a moderator variable to explore the relationship between climate change and the number of infectious disease cases [38]. However, whether environmental governance policies can improve public health is still highly controversial. Some researchers believe that the effectiveness of environmental governance is related to the enforcement capability of environmental protection agencies, a lack of which usually results in less effective environmental governance policies than expected [39]. Markus [40] found that although environmental governance policies can significantly improve air quality, such an improvement effect is still not enough to promote public health. To find new evidence for the impact of market-oriented environmental governance on public health, this paper, taking the pilot SO_2_ ETS in China as an example, constructs a DID model to estimate whether the marketization of air pollution control can bring about substantial health effects while improving air quality.

### 2.2. Theoretical Mechanism Analysis

Medical research reveals that long-term exposure to excessive SO_2_ will decrease human immune and defense functions, thereby increasing the incidence of cardiovascular diseases and respiratory infections such as chronic rhinitis, chronic bronchitis, and pneumonia. Limiting SO_2_ within a reasonable range through environmental governance policies can reduce the occurrence of related diseases and thus ensure public health. As a market-oriented means of environmental governance, ETS establishes legal SO_2_ emission rights and introduces a market mechanism to realize the purchase and sale of emission rights so as to control the total volume of SO_2_ emission and ultimately achieve the purpose of energy conservation, emission reduction, and environmental protection [41]. In essence, the ETS can reduce the prevalence of diseases related to air pollution and improve public health by redistributing pollution control tasks, forcing enterprises with low production efficiency to scale back production or to innovate technologically, optimizing resource allocation efficiency, promoting a green transformation of the economic development model, and reducing pollutant emissions [34,42].

According to the environmental justice theory, every individual or group in society should equally enjoy the right to health and welfare not to be infringed by the adverse environment, regardless of their status. However, due to factors such as income, education, occupation, etc., the problem of environmental inequality has become widespread [43]. The influence of the environment on health inequity mainly comes from two mechanisms: differential health effects and differential exposure levels [44]. Currently, scholars focus more on the first mechanism, such as health inequalities caused by social status, age, and gender, with little attention paid to the effect of differential exposure on health inequities. This paper discusses the health effect and internal mechanism of ETS on the differential exposure of environmental pollution caused by occupations and residential areas to capture the differential health effects realized by environmental governance.

The potential contribution of this paper lies in two aspects. On the one hand, this paper establishes a quasi-natural experiment of the ETS pilot to explore its effects and potential mechanisms on public health, providing new empirical evidence for the health effects of environmental governance from the perspective of market-oriented environmental governance. On the other hand, this paper fills the gap in the theoretical research on the heterogeneity of health effects of environmental governance on groups exposed to different pollution levels. Distinguishing from previous studies on age and gender heterogeneity, this study analyzes the heterogeneous health effects of environmental policies on different occupations and different regional populations due to different pollution levels based on occupational and demographic background information in the CHNS database.

## 3. Methodology and Data

### 3.1. Variables

Dependent variable. The dependent variable in this paper is public health. Some studies have found that environmental pollution is detrimental to the human respiratory system and heart [45]. Therefore, we use the incidence of respiratory diseases and heart diseases to measure health conditions that are sensitive to air pollution. In addition, we want to investigate whether ETS also provides other health benefits, and we choose the incidence of other diseases as one of the public health indicators. The public health data came from the China Nutrition and Health Survey (CHNS) database. Based on the survey results of “In the past four weeks, have you had any of the following symptoms?” in the CHNS database, individuals with fever, sore throat, cough, and asthma in the past four weeks are classified as having respiratory diseases; those with heart disease and chest pain are classified as having heart diseases; while those with diarrhea, dizziness and other symptoms are regarded as having other diseases.

Independent variable. The core independent variable in this paper is the interaction term between the ETS policy dummy variable and the policy implementation time dummy variable, indicating whether ETS is implemented in a province in different years. China first proposed the “4 + 3 + 1” pilot SO_2_ ETS policy in 2002, while China’s SO_2_ emission rights trading was still at the stage of “there are pilot rules and pilot zones exist, but few real transactions” [46]. It was not until 2007 that China formally established the first reserve center for emission trading. In the same year, the Ministry of Finance and the Ministry of Environmental Protection approved 11 pilot provinces, including Jiangsu, Zhejiang, Hubei, and Guangxi, marking the in-depth advancement of the pilot phase of ETS in China. Therefore, the existing literature generally takes 2002 [47,48] and 2007 [42,49] as the pilot time for ETS. Considering the actual implementation of ETS and its effect in China, this paper takes 2007 as the commencement of the SO_2_ ETS pilot and excludes three provinces of Shandong, Jiangsu, and Henan that were piloted in 2002. Based on the provinces polled in the CHNS database, we take the ETS pilot provinces of Hunan and Hubei as the experimental group and the other four provinces of Liaoning, Heilongjiang, Guizhou, and Guangxi as the control group. The interaction term takes the value of 1 if and only if the individual is from one of the two experimental group provinces and the survey wave is after 2007. Otherwise, it has a value of 0.

Control variable. The health condition of an individual is related to both individual characteristics and regional characteristics. Referring to the existing literature [36,50,51,52], at the micro level, this paper selects gender, urban–rural area, consumption of tobacco and alcohol, occupation, income, education level, body mass index, and medical insurance purchase of the individual as control variables. Since the health investment of an individual is usually determined by household income level rather than personal income level, we link individual health conditions with the per capita income of the family through household ID to measure income status. At the macro level, environmental governance efforts represented by regional per capita environmental governance investment, regional economic development measured by per capita GDP, urban greening denoted by per capita park green space, and regional population density are selected as regional control characteristics. The micro-control variables are from the CHNS, and the macro-control variables are collected from the China Environment Statistical Yearbook, China Health Statistical Yearbook, and the National Bureau of Statistics.

Mechanism variable. According to the mechanism analysis, EST can improve public health by lowering pollution levels; that is the total emissions of SO_2_ and NOx. Since the emissions of NOx in China were not officially counted until 2011, we chose the emissions of SO_2_ in each province to reflect environmental pollution and to empirically identify the above-mentioned potential influence mechanisms. The data of the mechanism variables in this paper are obtained from the China Environmental Statistical Yearbook. Table 1 shows the definition of the variables.

### 3.2. Statistical Analysis

The CHNS uses the multi-phase stratified cluster random sampling method to choose its subject. About 4000 households finally became the survey subjects through the level-by-level sampling of provinces, cities, counties, and communities. At the regional level, its ultimate sample covers urban and rural residents from nine provinces in China, including the east, middle and west; in terms of the level of economic development, low-income, middle-income, and high-income groups in each province are taken into account. The CHNS has performed ten surveys, the last of which was in 2015. Since the CHNS data are available over time intervals of two to four years, this paper mainly uses data from 2000, 2004, 2006, 2009, 2011, and 2015. After excluding samples with missing values, 20,734 observations are obtained. The statistical description of the variables used in this paper is shown in Table 2.

### 3.3. Model Setting

To explore the impact of ETS on individual health and its theoretical mechanism, this paper categorizes individual diseases into respiratory diseases (h_1_), heart diseases (h_2_), and other diseases (h_3_). If the individual suffers from category i disease, hi takes the value of 1, otherwise 0. Since the explanatory factors are discrete rather than continuous, this paper uses a binary logit model to construct the disease incidence, and for each j, the random utility of being the hi population is as follows:

(1) $$U_{\mathrm{jh}}=X_{j}\beta_{h}+\varepsilon_{\mathrm{jh}}$$

While U_jh_ is the health utility, X_j_ represents the individual’s gender, education level, and a range of other characteristics that affect health, β_h_ is the influence coefficient of X_j_ on U_jh_, ε_jh_ denotes the random disturbance term. Since ε_jh_ obeys two-point distribution, if we assume h_i_ = 1 indicates that the individual suffers from disease i, the following binary logit model of the disease probability for each individual can be established:

(2) $$P(h_{i}=1|X)=\frac{\exp\left(x_{j}\beta_{h}\right)}{1+\exp\left(x_{j}\beta_{h}\right)}$$

The DID method is frequently used to evaluate the effects of public policy implementation since it can successfully control regional differences before and after the implementation of policies, and exclude the effects of unobservable factors and time trends, thus separating the net effects of policy shocks. This paper aims to assess the health effects of market-oriented environmental governance by identifying the implementation effects of SO_2_ ETS, and the endogeneity problem can be effectively handled by using a DID model. The ETS started in 2007 is viewed as a quasi-natural experiment, with the two pilot provinces covered in the CHNS database as the experimental group and the remaining four provinces as the control group. Based on the research years in the CHNS database, three years of the non-pilot period (2000, 2004, 2006) and three years of the pilot period (2009, 2011, 2015) are selected to construct the following DID model:

(3) $${\mathrm{Health}}_{\mathrm{ijt}}=\beta_{0}+\beta_{1}{\mathrm{post}}_{\mathrm{it}}+\beta_{2}{\mathrm{treat}}_{\mathrm{ij}}+\beta_{3}{\mathrm{post}}_{\mathrm{it}}\times {\mathrm{treat}}_{\mathrm{ij}}+\varepsilon_{\mathrm{ijt}}$$

where i denotes the province, j represents individual, and t stands for year. ${\;\mathrm{Health}}_{\mathrm{ijt}\;}$ is public health condition measured by the incidence of respiratory diseases, heart diseases, and other diseases less correlated with air pollution; postit is the policy dummy variable of ETS, which takes the value of 1 for the post-implementation period (after 2007) and 0 for the pre-implementation period (before 2007); treatij is the province dummy variable, which takes the value of 1 if the individual belongs to the experimental group; and 0 otherwise; εijt denotes the random disturbance term.

In addition, considering that there are numerous variables affecting individual’s health, for the accuracy and rigor of the research results, this paper incorporates the aforementioned macro-level and micro-level control variables into Equation (3) and performs DID estimation again. The following model is obtained:

(4) $${\mathrm{Health}}_{\mathrm{ijt}}=\beta_{0}+\beta_{1}{\mathrm{post}}_{\mathrm{it}}+\beta_{2}{\mathrm{treat}}_{\mathrm{ij}}+\beta_{3}{\mathrm{post}}_{\mathrm{it}}\times {\mathrm{treat}}_{\mathrm{ij}}+\beta_{4}X_{\mathrm{ijt}}+\gamma_{i}+\delta_{t}+\varepsilon_{\mathrm{ijt}}$$

Xijt represents control variables, including micro-level control variables and macro-level control variables; γi is the province-fixed effect, δt is the time-fixed effect, and the remaining variables are consistent with those in Equation (3). β3 is the influence coefficient of ETS policy on the incidence of individual diseases, which is the focus of this study. If the coefficient is significantly negative, it indicates that ETS has an improvement effect on public health.

## 4. Empirical Results and Discussion

### 4.1. Baseline Results

Table 3 shows the binary logistic regression results based on Equation (1), in which columns (1)~(2), (3)~(4), and (5)~(6) are the regression results of the incidence of respiratory diseases, the incidence of heart diseases and the incidence of other diseases as the explanatory variables, respectively. Overall, the pilot ETS is beneficial to public health after controlling for the province-fixed effect and year-fixed effect, regardless of whether control variables are considered. The specific regression results are as follows: Firstly, the incidence coefficient of respiratory diseases is negative and statistically significant at the 10% level, indicating that individuals in the experimental provinces are less likely to suffer from respiratory diseases. Secondly, the regression results for the probability of heart diseases are not significant, i.e., there is no conclusive evidence that ETS has a positive effect in reducing the incidence of heart diseases. Finally, for the incidence of other diseases, the coefficient is significantly negative at the 1% level, suggesting that ETS lessens the risk of having other diseases that are less correlated with environmental pollution. This may be explained by the fact that air pollution can enter the bloodstream via the respiratory system and impair human immune function, thus making them more susceptible to other diseases. When air quality is improved, the risk of air pollution to immunity is correspondingly diminished, thereby declining the prevalence of other diseases.

In conclusion, the baseline results indicate that ETS reduces the incidence of both respiratory diseases that are susceptible to air pollution and other diseases that are less vulnerable to air pollution, which confirms that ETS has a significant improvement effect on public health. The key control factors include gender, urban–rural area, individual education level, lifestyle, and urban green space area, all of which affect public health. Consumption of alcohol is significantly and positively correlated with the incidence of respiratory diseases, while the per capita park green space has a significantly negative correlation with respiratory disease incidence.

### 4.2. Mechanism Analysis Results

Based on the study of Wen Zhonglin et al. [53], this paper constructs the following mediation effect model to verify whether SO_2_ has a mediating effect between ETS and public health:

(5) $${\mathrm{Pollution}}_{\mathrm{ijt}}=\beta_{0}+\beta_{1}{\mathrm{post}}_{t}+\beta_{2}{\mathrm{treat}}_{\mathrm{ij}}+\beta_{3}{\mathrm{post}}_{t}\times {\mathrm{treat}}_{\mathrm{ij}}+\beta_{4}X_{\mathrm{ijt}}+\gamma_{i}+\delta_{t}+\varepsilon_{\mathrm{ijt}}$$

(6) $${\mathrm{Health}}_{\mathrm{ijt}}=\beta_{0}+\beta_{1}{\mathrm{post}}_{t}+\beta_{2}{\mathrm{treat}}_{\mathrm{ij}}+\beta_{3}{\mathrm{post}}_{t}\times {\mathrm{treat}}_{\mathrm{ij}}+\beta_{4}{\mathrm{pollution}}_{\mathrm{ijt}}+\beta_{5}X_{\mathrm{ijt}}+\gamma_{i}+\delta_{t}+\varepsilon_{\mathrm{ijt}}$$

While pollution_ijt_ is the SO_2_ emission of province i in year t, other parameters are consistent with the above formula.

As shown in Table 4, ETS significantly reduces the emissions of SO_2,_ and this air quality improvement effect is conducive to reducing the incidence of respiratory diseases and other diseases, indicating that the emissions of SO_2_ have a mediating effect on the impact of ETS on public health.

### 4.3. Heterogeneity Results

The effects of environmental governance on public health, which are influenced by individual physiological characteristics and pollutant exposure levels, vary significantly across groups. Existing literature has explored the heterogeneous impact of environmental governance policies on health in various age and gender groups and found that the health effects of environmental governance are related to individuals’ physiological characteristics and immunity [32]. To determine the heterogeneity of health effects caused by varying levels of environmental pollution exposure, this paper investigates the heterogeneous health effects of ETS on different groups from occupational and urban–rural perspectives.

Firstly, in terms of occupation, different occupations are exposed to different degrees of environmental pollution and face different health risks. “Farmers, fishermen, hunters,” “drivers,” “skilled workers,” “unskilled workers,” and “service workers” are more likely to be exposed to pollution as a result of poor working conditions. To find out the different health effects on different occupational groups due to the implementation of environmental governance, we treat the above five occupations as near-pollution source workers. People of the other eight occupations who work indoors or in the administrative office are regarded as far-pollution source workers. Columns (1) and (2) of Table 5 show the regression results of health effects for near-pollution source workers and far-pollution source workers, respectively. We find that the implementation of ETS can significantly improve the health conditions of near-pollution source workers at the level of 10%, while the health improvement effects of far-pollution source workers are not significant. One possible reason is that the environmental improvement effect of EST is too weak to affect the health of far-pollution source workers. However, near-pollution source workers who are long exposed to air pollution are more sensitive to the changes of air quality. When air quality is improved, even modestly, the health risks of near-pollution source workers caused by environmental pollution will be substantially reduced.

Secondly, from the urban–rural perspective, differences in industrial structure and air quality between urban and rural areas in China lead to different levels of exposure to environmental pollution for urban and rural residents. In this paper, samples are divided into urban residents and rural residents to examine the heterogeneous effect of ETS implementation on the health of urban and rural residents. The regression results are shown in columns (3) and (4) of Table 5. The coefficient for rural individuals is significantly negative, while the coefficient for urban individuals is insignificant. In other words, ETS significantly improves the health of rural residents but does not affect the health condition of urban residents. The possible reason is that the main entities of SO_2_ emission trading in China are power plants, which are typically located in suburban or rural areas far from cities. The implementation of ETS reduces SO_2_ emissions from power plants, leading to significant improvements in air quality in suburban and rural areas, thereby improving the health of rural residents. It is not difficult to conclude from heterogeneity regression that ETS improves the health effects of some groups but not all groups. The possible reasons are that China’s ETS trading mechanism is still insufficient, the secondary market is not active, and inefficient market operation cannot support the perfect operation of ETS [49].

### 4.4. Robustness Checks

#### 4.4.1. Parallel Trend Test

**Figure 1:**
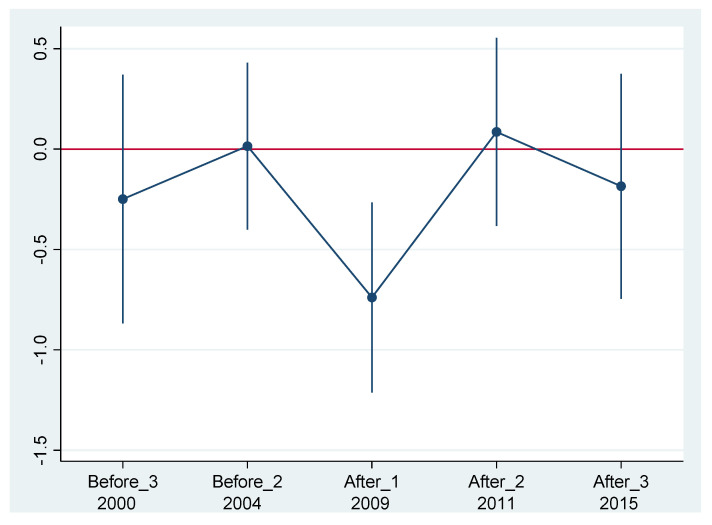
Parallel trend test results.

Since the DID method can effectively overcome endogenous problems, it has been widely applied in empirical studies. The critical premise of the DID method is that the experimental and control groups must satisfy the parallel trend assumption. Without the implementation of ETS, the trend of public health in pilot provinces should be similar to that of other provinces. Drawing on the parallel trend test of Beck [54], we set the treat value to 1 for the experimental group and 0 for the remaining four control provinces. The last period before the ETS implementation (2006) is named Before_1, and the first period following the ETS implementation (2009) is recorded as After_1. Similarly, the years 2000, 2004, 2011, and 2015 are recorded as Before_3, Before_2, After_2, and After_3, respectively. Figure 1 depicts the results dynamic regression results with the last period before ETS implementation as the base period. The confidence interval for the regression coefficients before the implementation of the ETS contains 0, but it does not contain 0 for the first phase following the implementation of the ETS, which verifies that the assumption of the parallel trend hypothesis is valid.

#### 4.4.2. Model Replacement

To further enhance the reliability of the empirical results, this paper replaces the binary logit model with a probit model [55] and tests the effect of ETS on public health based on Equation (4). From the estimated results in Column (1) of Table 6, we find that the signs and the significant levels of the estimated coefficients are consistent with the baseline results, validating the robustness of the baseline estimated results again.

#### 4.4.3. Placebo Test

There is no doubt that besides ETS, other policies or factors may interfere with public health. Therefore, it is necessary to further exclude the influence of such policies or factors. This paper advances the implementation of ETS to 2006 for a counterfactual test [56]. If the estimated coefficient is significantly negative, it indicates that the improvement in public health effect is likely to be caused by other policies or factors. Conversely, if the estimated result is not significant, it validates the previous conclusion. Column (2) of Table 6 demonstrates the case where ETS is implemented one year earlier and the test results are not significant, further confirming that ETS has a significant improvement effect on public health.

## 5. Implications

The additional health effects of ETS are supported by a series of robustness tests. However, our study also finds that ETS only improves the health of a small group, which means that the implementation effects of ETS are much lower than expected. One possible reason is that China’s ETS is still in the exploratory stage and has yet to form a standardized and unified market system. At present, the completed SO_2_ emission rights trading is still government-led, with an insufficient incentive for enterprises to participate independently, resulting in an inefficient market operation. Based on the above analysis, this paper proposes the following recommendations:

Firstly, a standard and long-term market mechanism is required to maintain the market-oriented principle and minimize government intervention. Therefore, it is essential to accelerate the formation of a reliable and unified trading system, break the current emission trading pattern dominated by government agencies, public institutions, and state-owned businesses, and allow the market to play a key role in resource allocation. Secondly, establishing a sound market price mechanism and information disclosure mechanism to protect the fairness of market transactions and reduce transaction costs, which helps motivate enterprises to actively participate in emission trading and activate the secondary market. Thirdly, the existing market trading pattern dominated by intra-provincial trading may lead to an imbalance between supply and demand for emissions trading, thus constraining the development of emission trading. Therefore, it is crucial to expand the transaction domain of emission rights, and promote cross-provincial and cross-industry emission trading.

## 6. Conclusions

This paper investigates the nexus of market-oriented environmental governance, environmental pollution, and public health by taking the establishment of ETS in 2007 as a quasi-natural experiment. According to the corresponding estimated results, the market-oriented ETS can decrease the incidence of public respiratory diseases and other diseases not directly related to environmental pollution. Further mechanism analysis results uncover that SO_2_ plays a mediating role in the implementation of ETS and public health. From the perspective of environmental equity, ETS can alleviate the problem of health inequity by reducing the health risks of vulnerable groups caused by long-term exposure to serious air pollution. Thus, it is feasible to improve public health and social welfare by promoting market-oriented environmental governance. Therefore, based on these findings, we propose some policy implications for promoting ETS and advancing the “Healthy China” strategy.

## Figures and Tables

**Table 1:** Definition of various variables.

Variable		Measurement	Variable	Definition
Dependent variable	Public health	Respiratory diseases	h_1_	Illness = 1; health = 0
		Heart diseases	h_2_	Illness = 1; health = 0
		Other diseases	h_3_	Illness = 1; health = 0
Independent variable		Implementation of ETS	did	Pilot = 1; non-pilot = 0
Control variables	Micro-control variables	Gender	gender	Male = 1, female = 0
		Urban–rural area	urban	Urban = 1, rural = 0
		Consumption of alcohol	drink	Yes = 1, no = 0
		Consumption of tobacco	smoke	Yes = 1, no = 0
		Occupation	ocup	Near-pollution source workers = 1; far-pollution source workers = 0
		Household income	lnhhincpc_~i	Ratio of net household income to household size
		Education level	edu	1–6 from elementary school to master’s degree, respectively
		Body Mass Index	bmi	BMI within reasonable range bmi = 1, otherwise bmi = 0
		Having medical insurance	ins	Yes = 1, no = 0
	Macro-control variables	Environmental governance efforts	lner	Regional per capita Environmental governance investment
		Economic development	lnragdp	Per capita GDP
		Regional greening	park	Per capita park green area
		Population density	lndensity	Ratio of regional population to regional area
Mechanism variable		emissions of SO_2_	lnSO_2_	emissions of SO_2_

**Table 2:** Statistical description.

Variable	N	Mean	SD	Max	Min
h_1_	20,734	0.054	0.227	1	0
h_2_	20,734	0.006	0.080	1	0
h_3_	20,734	0.098	0.298	1	0
bmi	20,734	0.692	0.461	1	0
lnhhincpc_~i	20,734	8.882	1.052	13.21	0.0520
smoke	20,734	0.360	0.480	1	0
drink	20,734	0.388	0.487	1	0
ins	20,734	0.586	0.493	1	0
gender	20,734	0.543	0.498	1	0
urban	20,734	0.286	0.452	1	0
edu	20,734	2.866	1.140	6	1
ocup	20,734	0.647	0.478	1	0
lner	20,734	2.520	0.978	4.267	0.390
lnragdp	20,734	9.307	0.652	10.60	7.909
park	20,734	7.741	2.620	12.94	2.450
lndensity	20,734	7.426	0.698	8.613	5.805

**Table 3:** Baseline empirical results of health effects.

	h_1_ Respiratory Diseases		h_2_ Heart Disease		h_3_ Other Disease	
	(1)	(2)	(3)	(4)	(5)	(6)
treat	0.309 **	0.210	−0.971 **	−3.591 **	0.156	0.00479
	(2.31)	(0.45)	(−2.42)	(−2.07)	(1.34)	(0.01)
post	1.222 ***	1.769	0.826 *	6.737*	1.097 ***	2.012 **
	(8.56)	(1.60)	(1.95)	(1.68)	(9.57)	(2.27)
did	−0.256 *	−0.283 *	0.285	0.668	−0.388 ***	−0.431 ***
	(−1.85)	(−1.69)	(0.62)	(1.12)	(−3.28)	(−3.01)
bmi		0.0471		−0.447 **		−0.0907
		(0.64)		(−2.19)		(−1.48)
lnhhincpc_~i		−0.0350		−0.000757		0.00944
		(−0.86)		(−0.01)		(0.30)
smoke		0.234 **		0.491		0.113
		(2.35)		(1.50)		(1.38)
drink		0.266 ***		−0.232		0.131 *
		(3.15)		(−0.82)		(1.80)
ins		0.112		0.719 **		0.164 **
		(1.16)		(2.38)		(2.04)
gender		−0.380 ***		−0.452		−0.323 ***
		(−3.70)		(−1.30)		(−3.72)
urban		0.577 ***		0.650 ***		0.583 ***
		(7.77)		(2.83)		(8.82)
edu		−0.130 ***		−0.660 ***		−0.279 ***
		(−3.58)		(−5.59)		(−8.65)
ocup		0.201 **		−0.356		0.103
		(2.52)		(−1.38)		(1.49)
lner		−0.129		−0.0861		0.0813
		(−1.03)		(−0.18)		(0.78)
lnragdp		0.365		−3.284		−0.296
		(0.48)		(−1.22)		(−0.47)
park		−0.118 **		−0.321		−0.105 ***
		(−2.54)		(−1.59)		(−2.78)
lndensity		0.0816		0.564 **		0.155 *
		(0.65)		(1.96)		(1.71)
Year fixed effect	yes	yes	yes	yes		yes
Province fixed effect	yes	yes	yes	yes		yes
Pseudo R^2^	0.00919	0.0143	0.00237	0.00558	0.0144	0.0245

Z-value in parentheses * *p* < 0.1, ** *p* < 0.05, *** *p* < 0.01.

**Table 4:** Mechanism analysis results.

	(1)	(3)
	h_1_ Respiratory Diseases	h_3_ Other Diseases
treat	0.0387	−0.181
	(0.04)	(−0.45)
post	2.254 **	2.636 ***
	(1.97)	(2.85)
did	−0.294 *	−0.456 ***
	(−1.75)	(−3.17)
lnSO_2_	−0.500 ***	−0.529 ***
	(−2.76)	(−3.34)
bmi	0.0467	−0.0917
	(0.64)	(−1.49)
lnhhincpc_~i	−0.0307	0.0141
	(−0.76)	(0.45)
smoke	0.233 **	0.113
	(2.34)	(1.38)
drink	0.266 ***	0.131 *
	(3.15)	(1.80)
ins	0.0942	0.145 *
	(0.98)	(1.79)
gender	−0.380 ***	−0.323 ***
	(−3.69)	(−3.72)
urban	0.571 ***	0.578 ***
	(7.70)	(8.74)
edu	−0.129 ***	−0.278 ***
	(−3.55)	(−8.62)
ocup	0.206 ***	0.108
	(2.58)	(1.56)
lner	−0.144	0.0830
	(−1.13)	(0.79)
lnragdp	0.325	−0.404
	(0.42)	(−0.64)
park	−0.139 ***	−0.130 ***
	(−2.86)	(−3.33)
lndensity	0.0171	0.0707
	(0.14)	(0.78)
Year fixed effect	yes	yes
Province fixed effed	yes	yes
Pseudo R^2^	0.0143	0.0243

Z-values in parentheses * *p* < 0.1, ** *p* < 0.05, *** *p* < 0.01.

**Table 5:** Heterogeneity results.

	(1)	(2)		(3)	(4)
	h_1_ Near-Pollution Source Workers	h_1_ Far-Pollution Source Workers		h_1_ Rural	h_1_ Urban

treat	0.508	−0.244	treat	0.639	−1.142
	(0.93)	(−0.26)		(1.03)	(−1.50)
post	0.887	2.734	post	−0.0181	5.997 ***
	(0.69)	(1.25)		(−0.01)	(3.31)
did	−0.348 *	−0.135	did	−0.391 *	−0.173
	(−1.71)	(−0.43)		(−1.88)	(−0.60)
bmi	−0.0509	0.230 *	bmi	0.0244	0.0245
	(−0.58)	(1.79)		(0.26)	(0.20)
lnhhincpc_~i	−0.0445	−0.0236	lnhhincpc_~i	−0.0331	−0.0339
	(−0.94)	(−0.31)		(−0.69)	(−0.47)
smoke	0.373 ***	−0.0166	smoke	0.140	0.458 ***
	(3.10)	(−0.10)		(1.08)	(2.90)
drink	0.102	0.535 ***	drink	0.0892	0.554 ***
	(1.03)	(3.60)		(0.83)	(4.01)
ins	0.0764	0.131	ins	0.173	0.00380
	(0.60)	(0.80)		(1.34)	(0.02)
gender	−0.413 ***	−0.182	gender	−0.229 *	−0.584 ***
	(−3.25)	(−1.09)		(−1.80)	(−3.37)
edu	0.419 ***	0.831 ***	urban	−0.186 ***	−0.0527
	(4.33)	(6.52)		(−4.02)	(−0.86)
ocup	−0.178 ***	−0.0575	edu	0.331 ***	0.0479
	(−3.99)	(−0.88)		(3.07)	(0.35)
lner	−0.121	−0.173	lner	−0.162	−0.102
	(−0.76)	(−0.80)		(−1.00)	(−0.50)
lnragdp	0.839	−0.229	lnragdp	1.274	−2.105 *
	(0.97)	(−0.14)		(1.29)	(−1.75)
park	−0.0935 *	−0.112	park	−0.106 *	−0.121
	(−1.69)	(−1.22)		(−1.78)	(−1.55)
lndensity	0.0470	0.152	lndensity	0.350 **	−0.136
	(0.31)	(0.69)		(2.03)	(−0.75)
Year fixed effect	yes	yes	Year fixed effect	yes	yes
Province fixed effect	yes	yes	Province fixed effect	yes	yes
Pseudo R^2^	0.0181	0.0148		0.0130	0.0191

Z-values in parentheses * *p* < 0.1, ** *p* < 0.05, *** *p* < 0.01.

**Table 6:** Robustness check results.

	(1)	(2)
	Model Replacement	Counterfactual Test
treat	0.105	−0.144
	(0.47)	(−0.31)
post	0.837	2.333 **
	(1.59)	(2.16)
did	−0.136 *	−0.0915
	(−1.67)	(−0.53)
bmi	0.0214	0.0437
	(0.61)	(0.60)
lnhhincpc_~i	−0.0142	−0.0394
	(−0.74)	(−0.97)
smoke	0.107 **	0.239 **
	(2.28)	(2.41)
drink	0.124 ***	0.265 ***
	(3.07)	(3.14)
ins	0.0548	0.112
	(1.17)	(1.16)
gender	−0.176 ***	−0.395 ***
	(−3.62)	(−3.85)
urban	0.281 ***	0.570 ***
	(7.65)	(7.69)
edu	−0.0625 ***	−0.0864 ***
	(−3.56)	(−2.79)
ocup	0.101 ***	0.217 ***
	(2.60)	(2.69)
lner	−0.0611	−0.165
	(−1.03)	(−1.27)
lnragdp	0.175	−0.0865
	(0.48)	(−0.12)
park	−0.0585 ***	−0.101 **
	(−2.64)	(−2.17)
lndensity	0.0414	0.0687
	(0.73)	(0.54)
Year fixed effect	yes	yes
Province fixed effed	yes	yes
Pseudo R^2^	0.0144	0.0142

Z-values in parentheses * *p* < 0.1, ** *p* < 0.05, *** *p* < 0.01.

## Data Availability

The data presented in this study are available on request from the corresponding author.
